# Crossing life-saving thresholds: learning-forgetting trajectories in secondary-school basic life-support training: project threshold-beat

**DOI:** 10.3389/fpubh.2026.1777690

**Published:** 2026-05-07

**Authors:** Raquel Cantón-Cortés, Pedro Fernández-Florido, Azahara Fernández-Carbonell, Francisco Manuel Parrilla-Ruiz, Gerardo Gómez-Moreno, María de los Ángeles Hernández-París, José Miguel Pérez-Villares, Antonio Cárdenas-Cruz

**Affiliations:** 1 Specialist in Family and Community Medicine, Emergency Department, Poniente University Hospital, El Ejido, Spain; 2 CriticalLab CTS 609 Research Group (PAIDI), TEC 23 IBS Granada Research Group, Granada, Spain; 3 Intensive Care Service, Hospital Universitario Virgen Macarena, Seville, Spain; 4 Intensive Care Service, Hospital University of Jaén, Jaén, Spain; 5 Family and Community Medicine, Emergency Department, Virgen de las Nieves University Hospital, Granada, Spain; 6 Department of Medicine, University of Granada, Granada, Spain; 7 PAIDI CTS 654 Research Group, Graduate in Medicine, University of Granada, Granada, Spain; 8 Department of Stomatology, Faculty of Dentistry, University of Granada, Granada, Spain; 9 Family and Community Medicine, Emergency Department, Torrecárdenas University Hospital, Almería, Spain; 10 Intensive Care Medicine Department, University Hospital Virgen de las Nieves Granada, Granada, Spain; 11 Intensive Care Unit, Virgen de las Nieves University Clinical Hospital, Granada, Spain

**Keywords:** basic life support, cardiopulmonary resuscitation, forgetting curve, learning curve, secondary education, stem education, threshold concepts

## Abstract

**Introduction:**

Basic life support (BLS) training in schools is a public health priority, but long-term retention of basic knowledge is poorly understood. The objective was to quantify the learning and forgetting curves of six threshold concepts (TC) of BLS in secondary school students and examine demographic moderators.

**Methods:**

A quasi-experimental design was implemented in six randomly selected schools (*N* = 459; age 12–15), whose students completed a validated 20-item multiple-choice test containing six BLS “key questions” at baseline (T0), immediately after instruction (T1), and 6 months later (T2). Friedman tests and mixed linear models assessed changes over time and interactions with gender, grade, and school location.

**Results:**

The mean TC score increased from 40.5% (T0) to 67.3% (T1) (Δ = 26.8%, *p* < 0.001; *d* = 1.22) and decreased to 55.1% (T2) (-18.1% from T1; *p* < 0.001; *d* = 0.63). Females and 4th-year students retained significantly more knowledge (β = 0.71 ± 0.18 and β = 0.80 ± 0.17, respectively; both *p* < 0.001). Recognition of agonal breathing showed the most pronounced forgetting gradient (-27%), while time-critical remained stable (-9%).

**Discussion:**

BLS competence depends on a small set of transformative concepts that decay at differential rates. Quarterly micro-refreshers focused on agonal respiration recognition and AED sequencing are recommended to consolidate learning pathways beyond the liminal phase.

## 1 Introduction

Cardiopulmonary arrest (CPA) is defined as the sudden, unexpected, and potentially reversible cessation of cardiopulmonary functions, and is a time-dependent condition (1–3). It is considered by the World Health Organization to be a serious public health problem due to its high morbidity and mortality rates (4–6); cardiac-related CPR is the leading cause of death in industrialized countries (7). In Spain, there are approximately 24,500 cases of CPR per year (1), with the majority (around 80%) occurring outside of hospitals (4). It is estimated that more than 70% of these CRPs are witnessed (8), yet in less than 30% of cases do these witnesses initiate cardiopulmonary resuscitation (CPR) before the arrival of emergency medical services (EMS) (4, 7, 9–11). Some of the reasons for this are not knowing how to recognize a cardiac arrest, lack of CPR knowledge, and fear of contracting an infection, causing injury, or making mistakes (6). These figures have remained stable over time, with overall survival rates for cardiac arrest hardly changing in the last two decades, standing at around 10% in both the US and Europe (5, 7, 11). However, it is known that early initiation of CPR by witnesses can double or triple the probability of survival (1, 7, 9). Thus, it has been demonstrated in other countries, such as Japan and Sweden, where basic CPR training is widespread, that training the general population leads to a significant increase in the rate of resuscitation by bystanders (3, 12).

Cardiopulmonary resuscitation (CPR) is a series of sequential actions designed to replace the cardiopulmonary functions that the patient has lost (1, 2); while basic life support (BLS) is a broader concept that includes preventing cardiac arrest as far as possible, detecting it when it occurs, early activation of emergency medical services, initiation of basic CPR measures, and use of an automated external defibrillator (AED) (1–3).

The chain of survival is understood to be the set of actions that, when performed in an orderly manner and at the right time, have been shown to reduce mortality in patients who have suffered a CPA (2). Several studies targeting the general population show a lack of knowledge about the first two links in the chain (1. early recognition + activation of EMS and 2. early initiation of CPR), and therefore the need to improve cognitive, procedural, and attitudinal skills related to BLS in order to improve survival from out-of-hospital cardiac arrest (3, 7, 13).

It is estimated that at least 15% of the general population needs to be trained in BLS in order to achieve a significant improvement in survival rates, and voluntary training of the population is not sufficient to achieve this goal (5, 6). Both the American Heart Association (AHA) and the European Resuscitation Council (ERC) advocate for the initiation of BLS training at school age, as this is an easily accessible population given that primary and secondary education is compulsory, they are interested and willing to perform CPR maneuvers, they can learn more quickly and are able to retain what they have learned much better, and, in addition, this type of intervention is well received by the educational community (5–7, 14).

In Spain, Royal Decree 126/2014 (15) and 1105/2014 (16), which establish the training itineraries for primary and secondary education, respectively, include first aid training but do not establish the mandatory content to be taught, so BLS and CPR are not mandatory in school training. However, in an attempt to include BLS training at school age, various institutional initiatives and projects have been carried out in schools (10), such as: the “Cervantes Model in Granada,” the “Alertante Program” in Madrid, “The ABCs that save lives” by the Government of Navarre, “Health emergencies in schools for educational centers” by the Basque Government, the “Pilot Plan” of the Catalan Resuscitation Council together with the Ministry of Education and Health with “spiral training,” “CPR in the Classroom” in Lugo, the “PROCES (Cardiopulmonary Resuscitation Program for Secondary Schools)” in Barcelona, etc. (1, 3, 17).

However, despite the proliferation of basic life support training programmes aimed at the school population, the available evidence shows that cognitive retention is heterogeneous and that certain content has more pronounced forgetting trajectories. This limitation suggests that not all elements of the curriculum have the same structural weight in the consolidation of learning. From this perspective, it is pertinent to explore whether such difficulties could be explained not only by methodological factors or the frequency of recycling, but also by the epistemological nature of the content taught.

In this sense, the theory of threshold concepts proposed by Meyer and Land describes certain knowledge as transformative, integrative and irreversible, insofar as it reconfigures the learner's understanding and allows access to a new way of thinking within a discipline (18). Applied to the field of basic life support, this approach allows us to hypothesis that certain elements - such as the recognition of agonal breathing, the critical sequencing of actions or the prioritization of high quality chest compressions - could constitute conceptual nodes whose adequate internalization conditions the integration of the rest of the algorithm. Thus, the variability observed in the learning and forgetting curves could be interpreted as a reflection of different degrees of crossing the conceptual threshold, rather than as simple memory loss.

In Meyer and Land's framework, a threshold concept differs from a core concept in that it acts as a ‘portal' that transforms understanding and action, integrates previously separate ideas, and tends to consolidate as a mental framework that is difficult to reverse (18–24). In sequential and time-dependent clinical domains such as basic life support, such thresholds can manifest themselves in operational form (decision ru-les and algorithmic sequences). Based on this approach, we selected six critical cog-nitive elements (key questions) that we consider aligned with threshold criteria (transformation, integration and irreversibility) and analyzed them as candidates for threshold concepts to study their learning and forgetting trajectories.

The objective of this study is to determine the degree of acquisition of cognitive skills within a life support training program, as well as to determine the frequency with which this type of training activity should be carried out based on the knowledge acquired, based on the study of the forgetting curve and the impact of threshold concepts on this type of training.

## 2 Material and methods

### 2.1 Design and procedure

A quasi-experimental study was conducted with repeated measures at three time points. At T0 (baseline), participants completed the knowledge questionnaire (20 items) prior to training. They then received a standardised BLS educational intervention that combined scripted pre-briefing, brief theory block, hands-on CPR and AED stations with deliberate practice, integrated scenarios and structured debriefing. At T1 (immediate post-intervention), the questionnaire was repeated to estimate acquisition. At T2 (6-month follow-up), the questionnaire was administered again to analyze retention/forgetting. The intervention was implemented with a closed teaching package and standardisation of instructors, including calibration session and fidelity of implementation checklist.

The 6-month time frame was chosen based on what has been established in scientific literature. Thus, although various scientific societies such as the AHA (American Heart Association) and the ERC (European Resuscitation Council) recommend annual refresher training to mitigate the forgetting curve, the current literature does not provide a clear turning point at which knowledge and skills in BLS begin to decline (6, 7, 12, 25–32); however, some studies suggest that this occurs between 3 and 6 months (33, 34). In this regard, different authors disagree on the frequency of such refresher training. Cárdenas Cruz et al. recommend refresher training after 12 months (25); Saad et al. proposed a retraining frequency of 18–24 months for retention to be greater than 70% or every 12 months for it to be greater than 80% (35); Arriola Infante et al. showed that in the three main techniques of BLS (basic CPR, defibrillation, and airway management), the forgetting curve was even faster, with statistically significant differences observed after 3 months (26); other authors maintain that theoretical knowledge seems to be retained for up to 18 months, while practical skills are retained for 6–9 months. The timing was chosen based on what has been established in the scientific literature, where the retention of cognitive skills begins to decline after 6 and 7 months (forgetting curve) (26).

Of the 20 items on the knowledge questionnaire, six referred to critical actionable cognitive elements of BLS:

- Safety and initial assessment. The “key message” conveyed was that first, the safety of both oneself and the subject in question must be ensured, and then the response and breathing must be checked, which should take no more than 10 s. During the training activity, the teacher demonstrated to the students how to safely approach and check for response and breathing using a case study similar to their own situation. The students then worked in pairs to perform the CHECK-CALL sequence. They were assessed on their ability to assess the subject in less than 10 s and activate the emergency medical services and third parties.
- Hands in the center of the chest. The “key message” was conveyed that in order to perform effective, high-quality compressions, the hands must be placed in the center of the chest. During the training activity, the teacher demonstrated where to place the hands using a simple anatomical reference, after which the student performed 10–15 guided compressions. The correct position of the hands and allowing the chest to re-expand between compressions were assessed.
- Complete sequence of the algorithm. The sequence “recognize-call-compress-AED” without critical gaps was conveyed as the “key message.” To this end, the teacher presented several brief integrated scenarios relevant to schoolchildren, in which the students took turns participating in different roles. The following were assessed: not omitting any steps in the algorithm, activating the emergency system, and minimizing pauses in chest compressions.
- Breathing in a maximum of 10 s. The “key message” conveyed was that in the event of abnormal breathing, one must act quickly and begin CPR. To demonstrate agonal breathing, the teacher showed a short video and simulated this type of breathing so that the students could identify it, and then gave them decision-making exercises. A correct decision made in a timely manner was valued.
- Continuous, high-quality compressions. The “key message” conveyed was that the most important thing is to compress “hard and fast, without interruption.” To this end, the instructor first demonstrated the rhythm and changes in the rescuer to perform continuous, high-quality compressions, and then the students practiced and developed these skills in 2-min blocks on a mannequin. The frequency of compressions and pauses within the range were evaluated.
- Severe choking. The “key message” conveyed was that when a person cannot cough or speak, the situation is serious and action must be taken. The instructor initially demonstrated the procedure to follow in the event of choking, and then the students performed a supervised role-play in which they acquired the necessary skills to handle choking. The ability to identify the severity and perform the sequence correctly was assessed.

During the training, the six critical cognitive elements were reinforced with microlearning and feedback after each student's intervention and at the end of the session. Table 1 shows the operationalization pattern described for each of these elements.

**Table 1:** Operationalization pattern for critical actionable cognitive elements.

Key element	Key message (1 sentence)	Teaching activity	Learner practice	Performance criteria	Linked assessment
Safety + initial assessment	Safety first; then check response and breathing ≤ 10 s.	Scripted demonstration with school case	CHECK-CALL sequence in pairs	Does not exceed 10 s; activates help	Key item + checklist
Hands on center of chest	Hands go to the center of the chest to effectively compress.	Demonstration with simple anatomical reference.	10–15 guided compressions per student	Correct position and re-expansion	Key item + checklist
Complete algorithm sequence	Recognise-call-compress-DEA: no critical jumps.	Short integrated scenario	Turn-based scenario (roles)	No omission of activation; minimises pauses	Key item + checklist
Breathing in ≤ 10 s	If not breathing normally, decide quickly: start CPR.	Short video + demonstration of agonal breathing	Decision exercises (cards)	Correct decision in time	Key item
Continuous quality compressions	The most important thing is to compress hard and fast, without interruptions.	Pace training and rescuer changes	2 min blocks on manikin	Frequency/pauses within range	Key item + checklist
Severe choking	If unable to cough or speak, it is severe: act.	Scripted choking demonstration	Supervised role-play	Identify severity and correct sequence	Key item

### 2.2 Reporting guidelines

This manuscript has been prepared following the recommendations of the GREET (Guideline for Reporting Evidence-based practice Educational interventions and Teaching) guide, available on the EQUATOR network, to ensure transparency and replicability of the educational intervention. Given the quasi-experimental nature of the design, reporting elements recommended by TREND for non-randomised studies were also incorporated. The completed GREET checklist is included as Supplementary material.

#### 2.2.1 Context

In Spain, basic education is compulsory and free and comprises primary education and compulsory secondary education (ESO). Primary education consists of 6 years between the ages of 6 and 12 and ESO consists of 4 years between the ages of 12 and 16, with the possibility of extending to 18 if the student repeats a year. After ESO, pupils can go on to Bachillerato (2 years) or to intermediate vocational training. The system is regulated by the Organic Law on Education (LOE) and its amendment, the LOMLOE, and is a decentralized model in which the Ministry sets the minimum education standards and the Autonomous Communities develop the curriculum.

Six secondary schools in Granada and its metropolitan area were selected. Based on their location, the sample was divided into schools belonging to the city of Granada and schools in the metropolitan area, with three schools randomly selected from each group (El Carmelo, Ganivet, and Padre Manjón secondary schools as city schools; and Alfacar, Atarfe, and Pulianas secondary schools as metropolitan area schools).

#### 2.2.2 Participants

Initially, all students in the first year of secondary school (12 years old) and the fourth year of secondary school (15 years old) from the randomly selected schools in the city of Granada and its metropolitan area (mentioned in the previous subsection) were included, with a total initial sample of 459 students.

Those students who did not complete the evaluation questionnaire at any of the three times it was to be completed (prior to training, immediately after training, and 6 months after the intervention) were excluded, leaving a final sample of 420 students.

#### 2.2.3 Trainers

The training programme was designed, implemented and delivered by instructors in basic life support and advanced life support, all of whom belong to the National CPR Plan of SEMICYUC (Spanish Society of Intensive, Critical and Coronary Care Medicine), which guarantees the homogeneity of the training process since all the participating teachers had received the same type of previous instruction. The National CPR Plan belongs to the Spanish CPR Council, which is the body in charge of representing the European Resuscitation Councial (ERC) in Spain, so we have all the guarantees for the performance of training actions in life support at any level.

In addition, all instructors were coordinated by an instructor/director responsible for all aspects of logistics, order, academics and design of the training action.

To standardise instructors, a closed teaching package (slides, demonstration script, station che-cklist and debriefing guide), a preteaching calibration session and a fidelity checklist applied du-ring implementation were used to standardize instructors.

#### 2.2.4 Intervention

The training was structured in scripted pre-briefing, short theory block (with the help of slides and a short video), practical stations with deliberate practice (CPR/AED) and integrated scenarios (with the help of a training mannequin and AED), and a structured debriefing.

Table 2 shows an operational description of the intervention, which allows for direct replicability while maintaining consistency across educational centers.

**Table 2:** Operationalisable description of the intervention.

Component	Objective	Format	Duration	Materials	Standardization
Pre-briefing	Aligning objectives and security	Structured script	5–10 min	Slides + script	Common verbatim text
Theoretical block	Key concepts SVB	Interactive micro-lesson	20 min	Presentation + short video	Closed slides
CPR practice	High quality compressions	Rotary stations	30 min	Adult manikin + AED training	Performance checklist
Integrated scenario	Full algorithm application	Short simulation	15 min	Single script + timer	Standardized sequence
Debriefing	Consolidate decision rules	Structure 3 phases	10 min	Question guide	Fixed template
Micro-refresher	Spaced active retrieval	Digital microlearning	5–10 min/unit	Capsules and questions	Closed content bank

For theoretical teaching, a standardized presentation (slides) in PPT format was used, in accordance with the 2015 ERC recommendations, which allowed for an introduction to the BLS algorithm and reinforced key messages. In addition, to ensure consistency in the messages, timing, and examples given by the instructors, an instructor's teaching guide (script) was used. Short capsules and review questions in digital format (QR/LMS) were used for spaced reinforcement and active recall.

In the practical workshops, Laerdal adult CPR training manikins were used for the acquisition and training of BLS skills. On the other hand, a training AED (automated external defibrillator; without discharge) was used for training in the safe sequence of use and minimization of pauses during defibrillation. Both the manikins and the AED were provided by the Department of Medicine at the University of Granada.

Theoretical knowledge was assessed using a knowledge questionnaire (20 items) that the students completed on three occasions (before the training, immediately after the training, and 6 months after the training). Practical skills were assessed using a performance checklist (CPR/AED), which allows for feedback and standardizes practical assessment.

Finally, a structured debriefing guide was used to consolidate learning and promote metacognition after the practical session.

Table 3 provides a more visual overview of the materials used.

**Table 3:** Teaching materials used.

Category	Material/resource	Format	Use in the session
Theoretical teaching	Standardized presentation (slides)	PPT/PDF	Introduction to the SVB algorithm and key messages
Theoretical teaching	Instructor's teaching script	Document	Ensures homogeneity of messages, timing and examples
Microlearning	Short capsules + retrieval questions	Digital (LMS/QR)	Spaced reinforcement and active retrieval
Skills	CPR (adult) training manikin	Physical	Deliberate practice of compressions
Defibrillation	Training AED (non-shock)	Physical	Safe sequence of use and minimisation of pauses
Assessment	Performance Checklist (CPR/AED)	Document	Standardises practical assessment and feedback
Evaluation	Knowledge questionnaire (20 items)	Document	Pretest, posttest and follow-up
Debriefing	Structured debriefing guide	Document	Consolidation and metacognition after practice

The curriculum was aligned with current recommendations for resuscitation and BLS education, which at the time of the teaching intervention were the 2015 recommendations, adopting a school-based approach (“Kids Save Lives”), for which the language level, teaching sequence, and dosage were adapted to the secondary education context. Thus, a strategy of “adoption with contextual adaptation” was chosen, in which the structure of the algorithm (safety-response-breathing-activation-compressions-AED) and quality standards (e.g., high-quality compressions and minimal interruption) are maintained, but the examples and narratives are adjusted to a school context, the timing and size of groups (ratio in practical workshops 8:1 students:instructor), the degree of theoretical depth, and the microlearning format to facilitate spaced practice.

It should be noted that the study does not aim to validate a new “proprietary” curriculum, but rather to evaluate a school implementation that can be standardized in guidelines, focusing on six critical elements of performance.

#### 2.2.5 Schedule

Two sessions were held. The first lasted 150 min, during which the schoolchildren began by completing the 20-item knowledge questionnaire (T0). This was followed by 120 min of theoretical and practical training, and finally, the questionnaire was repeated (T1).

The second session took place 6 months after the first and lasted 30 min, during which the students completed the theoretical knowledge questionnaire for the third time (T2). Afterwards, any questions that may have arisen during that time period were answered.

#### 2.2.6 Evaluation

A multiple-choice assessment (Table 4) was designed as a measurement tool, consisting of 20 questions on BLS concepts with five possible answers and one correct answer. This assessment included six “key” questions on the knowledge considered essential for learning the maneuvers properly, which formed the basis for the subsequent definition of the TC. These questions were selected based on their importance: two dealt with the basic sequence of performing the maneuver, two were practical cases to assess its application in real life, and two dealt with the importance of time and chest compressions.

**Table 4:** Survey for evaluating theoretical knowledge based on defined threshold concepts.

1) If you find yourself alone with a person lying on the ground, what is the first thing you should do before starting CPR (cardiopulmonary resuscitation)? ^*^ a) Place the person with their feet elevated b) Search their clothes for their family's phone number c) Pour cold water on their face to see if they react d) Place them in the recovery position e) Check that the victim and the rescuer are safe and immediately place the patient in the CPR position (supine—face up—with arms close to the body) and check for consciousness by shouting and shaking them
2) Indicate which statement is false: a) **Most cardiac arrests occur in hospitals**. b) Most cardiac arrests occur outside of healthcare settings. c) Most cardiac arrests occur outside of healthcare settings and are itnessed. d) Most cardiac arrests occur outside the healthcare setting, are witnessed, and usually occur in the patient's home. e) The percentage of the healthcare population trained in life support in our country is very low.
3) Chest compressions are performed by compressing the chest with arms extended and the heel of the hands applied to: ^*^ a) The left nipple b) **The center of the chest, on the sternum, and between the two nipples** c) The thorax, regardless of location d) The upper third of the sternum, in the midline e) The left rib cage (ribs)
4) The following may be used in basic life support: a) **Mouth-to-mouth barrier devices** b) Guedel airways c) Self-inflating bag with mask d) Laryngoscope e)No devices may be used in basic CPR, only our hands and mouth
5) CPR maneuvers cannot be interrupted in the following circumstances: a) If the patient shows signs of life b) When we know with certainty that it is materially impossible to receive help from emergency teams within 10 min of starting CPR c) **If after the first 5 min of basic CPR we do not obtain any response** d) If the patient shows clear signs of life after the first shock with the semi- automatic defibrillator e) When the rescuer is exhausted during basic CPR
6) When faced with a patient who collapses in front of us and who, when we approach and use the “shout and shake” maneuver, does not respond, what should be the first action to take? a) Call the emergency services immediately by dialing 911, as the patient may be in cardiac arrest. b) Place the patient in the recovery position. c) **Open the airway using the head-tilt chin-lift maneuver and then check whether they are breathing using the look, listen, and feel maneuver**. d) Leave them alone, they may just be tired. e) Immediately begin CPR techniques: chest compressions and mouth-to- mouth resuscitation
7) With regard to the use of semi-automatic external defibrillation (SAED) during a cardiac arrest, it is false that: a) The use of SAED can restore spontaneous circulation in a patient with cardiac arrest due to a shockable rhythm b) **Only doctors and nurses can use these devices** c) It is a device that can be used by anyone with the appropriate level of training. d) They have a positive impact on the survival of patients in cardiac arrest. e) They must be installed in any public place with an annual attendance of more than 5,000 people.
8) Define the correct sequence of actions to take if a man in his 50s collapses in front of you: ^*^ a) The first thing to do is to give him a precordial thump (punch him in the chest with all your strength), as this is the best way to reverse a cardiac arrest outside a hospital setting. b) Call 911, place the patient in the recovery position, and wait. c) **Check for unconsciousness (no response), check for breathing, and if he is not breathing, call for help and activate the emergency medical system, check if there is an AED in the vicinity, and begin basic CPR with a 30:2 ratio while waiting for the help you have requested**. d) check for unconsciousness, and if the patient is unconscious, diagnose cardiac arrest and begin life support measures e) The best thing we can do is cross to the other side of the street and stay out of trouble
9) After confirming that a patient is unconscious using the “shout and shake” maneuver, we have opened the airway using the head-tilt chin-lift maneuver and then checked for breathing using the look, listen, and feel maneuver, and finally confirmed that the patient is not breathing. What would be the next step? a) Begin chest compressions and mouth-to-mouth ventilation with a 30:2 ratio b) Begin chest compressions and mouth-to-mouth ventilation with a 5:1 ratio c) Begin chest compressions only d) **Call for help, activate the emergency medical system by calling 911, request an AED if available, and begin CPR** e) Place the patient in the recovery position
10) Which of the following factors related to circulatory support through external cardiac massage has the greatest influence on patient survival: a) Combining chest compressions with mouth-to-mouth ventilation b) Depressing the sternum exactly 8 cm c) Depressing the sternum exactly 10 cm d) Performing compressions at a rate of more than 80 compressions per minute e) **Performing continuous, high-quality chest compressions**
11) When is the appropriate moment during basic life support to request a semi- automatic external defibrillator (SAED)? a) The SAED is a medical device and therefore does not fall within the scope of BLS. b) **Once we have checked that the patient is unresponsive and not breathing, and we have activated the emergency medical services by calling 911, we will request the AED if it is available**. c) When we have been performing basic CPR with chest compressions and mouth-to-mouth ventilation for at least 10 min. b) When the emergency medical services arrive. e) The AED can only be used by healthcare personnel.
12) What is the name of the technique used to open the airway in basic life support? a) Jaw thrust maneuver b) Claw maneuver c) Triple airway opening maneuver d) Kotcher maneuver e) **Head-tilt chin-lift maneuver**
13) How long can we take to assess whether the patient is breathing using the “look, listen, and feel” method? ^*^ a) 10 min b) **10 s** c) 1 h d) 15 s e) 15 min
14) When there are two rescuers trained in basic life support who are going to perform CPR, how should they proceed? a) **The rescuers perform basic CPR techniques (chest compressions and mouth-to-mouth ventilation) taking turns every 2 min**. b) One rescuer performs chest compressions and the other performs mouth- to-mouth ventilation, taking turns as they become tired. c) It is not recommended to perform CPR with two rescuers. d) The first rescuer performs basic CPR techniques (chest compressions and mouth-to-mouth ventilation) until they become tired, and then the second rescuer performs the same techniques until they become exhausted, and they continue to alternate in this manner. e) One rescuer performs chest compressions and mouth-to-mouth ventilation, and the other rescuer calls for help, dials 911, and goes to get the AED (that is the end of their role)
15) Of all the techniques included in basic life support, which of the following has the greatest overall impact on the survival of patients undergoing CPR? ^*^ a) Calling for help b) Mouth-to-mouth ventilation c) Head-tilt chin-lift maneuver d) **Continuous, high-quality massage** e) Look, listen, and feel maneuver
16) When should emergency medical services be activated for a child in cardiac arrest if there is only one rescuer? a) After opening the airway b) After checking for breathing and giving five breaths c) After checking for “signs of circulation,” including lack of pulse d) **After 1 min of breaths and chest compressions** e) After 5 min of CPR
17) In a case of partial choking where the victim can cough and speak, what should we do? a) Give them 5 back blows b) Perform the Heimlich maneuver (5 abdominal thrusts) c) Perform 5 chest compressions d) Give them 5 back blows, followed by 5 chest compressions e) **Encourage them to cough**
18) If an adult has suffered an airway obstruction due to a foreign body and is unconscious on the floor, where should we place our hands to perform abdominal thrusts, also known as the Heimlich maneuver? a) On the chest, in the lower third of the sternum b) On the abdomen, below the navel c) On the abdomen, between the navel and the xiphoid process (at the pit of the stomach) d) On the lower abdomen e) **Abdominal thrusts should not be performed; instead, CPR should be initiated with chest compressions and mouth-to-mouth ventilation**
19) When should the Heimlich maneuver or abdominal thrusts be used? ^*^ a) When the victim loses consciousness b) When the victim is a child c) When the victim coughs d) When the victim vomits e) **When the choking victim cannot speak or cough, and the obstruction persists after 5 back blows**
20) During basic CPR in infants (first year of life): a) **Before starting chest compressions, administer 5 rescue breaths**. b)When only one rescuer is available, the first thing to do is call 911. c) Compressions should be administered by placing both hands in the center of the chest. d) Assessment of breathing should not take more than 5 s under any circumstances. e) Chest compressions should depress ½ the width of the chest

Key questions are those marked with. (^*^)Correct answer marked in red.

The internal consistency of the 20-item questionnaire (dichotomous scoring: 1 = correct, 0 = incorrect) was assessed using the Kuder-Richardson coefficient (KR-20), appropriate for instruments with dichotomous items. A value of KR-20 = 0.87 was obtained, indicating a good-high internal reliability, suitable for the assessment of longitudinal changes in basic life support knowledge.

#### 2.2.7 Analysis

All data were collected and evaluated anonymously, so that the identity of the individuals to whom each piece of data belonged could not be known. To perform the data analysis, a database was created in the SPSS 20.0 statistical analysis program. The variables taken into account were: age, sex, and location (characterized as dichotomous quantitative nominal variables), total number of correct answers obtained in each test (discrete quantitative variable), and correct answers for each question (dichotomous numerical nominal variable). Linear mixed models were used for repeated measures, with time (T0, T1, T2) as a fixed effect and a random intercept per participant (and per class/center if applicable), estimating pre-specified contrasts (T1 vs. T0; T2 vs. T1; T2 vs. T0) and reporting estimates, 95% CI and effect sizes. Sensitivity analyses were performed for missing data and alternative correlation structures.

The model is specified as:

- Dependent variable: total score (hits) or percentage.
- Fixed effects: time (T0/T1/T2), gender, course, location and their interactions with time.
- Random effect: intercept per participant (and, if appropriate, covariance structure for repeated measures).
- Contrasts: EMMs (estimated marginal mean) (a) changes T0 → T1, T1 → T2, T0 → T2 and (b) differences between groups at each time.
- Presentation: difference, 95% CI and multiplicity-adjusted p where appropriate

## 3 Results

The epidemiological characteristics of the students are shown in Table 5, where the 420 students who completed the study are classified according to the variables of age, sex, and location of their educational center.

**Table 5:** Distribution of the sample variables.

School year	*N* (%)
4th CSE	192 (45.7)
1st CSE	228 (54.3)
Gender	
Female	208 (49.5)
Male	212 (50.5)
Location of the school	
Center	220 (52.4)
Periphery	200 (47.6)

**Figure 1:**
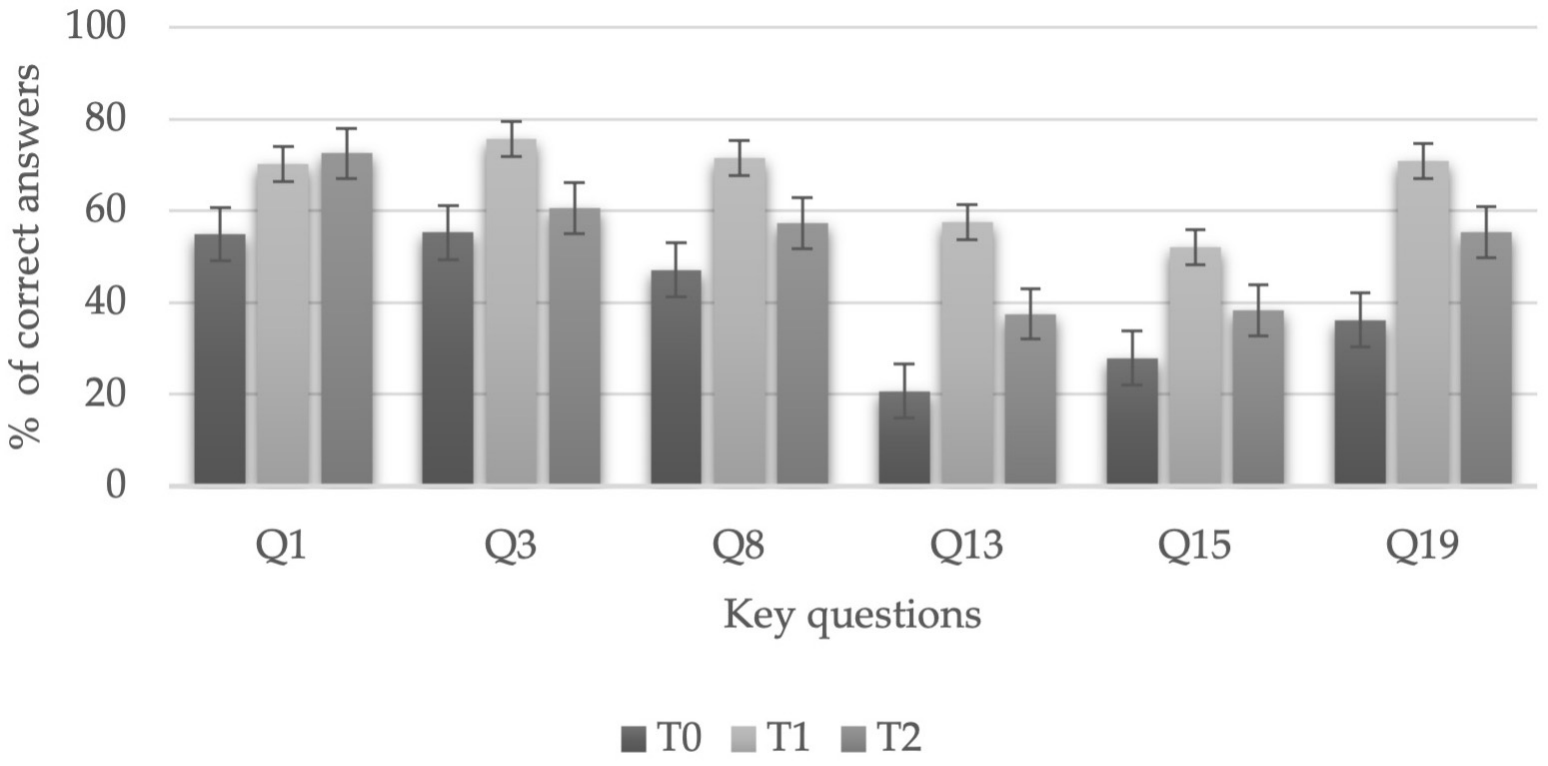
Precentage of correct answers to the ‘'key” questions in the 3 evalutions.

Figure 1 shows the percentage of students who answered each “key” question correctly in the three assessments; it can be seen that the average percentage increased between T0 (40.51%; *p* < 0.001) and T1 (67.3%; *p* < 0.001). After 6 months, at T2, this average decreases by 10% compared to that obtained after training, to 57.25% (*p* < 0.001). A detailed study of these questions shows that the above trend is common to all of them except for the first question, which refers to the BLS algorithm, and in which the percentage of correct answers increases in the three tests carried out (54.9, 70.2, and 72.5%).

Estimates derived from the repeated measures mixed model described in Methods will be prioritized because (i) it incorporates the T0-T1-T2 within-subject correlation, (ii) it allows for the inclusion of time × group interactions, and (iii) it more appropriately handles losses to follow-up (under MAR assumptions).

If we take into account the demographic variables studied in relation to the prior assessment of knowledge, statistically significant variations were only observed in the school year, with students in higher grades and therefore older students answering more questions correctly (Table 6). Therefore, “center-periphery = 0.377,” which means that, at the time of the assessment, students in the center of Granada obtained 0.377 more correct answers (on average) than those in the periphery. As for the school year, “4th CSE - 1st CSE = 0.838,” which means that, in the average score obtained at that time, 4th CSE obtained 0.207 more correct answers (on average) than 1st CSE. Finally, in relation to gender, “women - men = 0.207” means that, at the time of the assessment, women obtained 0.207 more correct answers (on average) than men.

**Table 6:** Comparison of results in the initial evaluation (T0) according to the 3 variables analyzed.

Comparison (first – second)	Difference	CI 95% (lower; upper)	P
Center–Periphery	0.377	0.076–0.830	0,102
4th CSE−1 st CSE	0.838	0.390–1.286	0,000
Female–Male	0.207	0.247–0.660	0,371

The values indicated correspond to contrasts of estimated marginal means (EMMs).

The level of knowledge acquired is significantly related to the age/grade of the student and to gender. In the exam taken immediately after the theoretical-practical intervention, we observed that women, students in the higher grade, and schools in the city of Granada (the latter with less statistical significance) acquired a higher level of knowledge (Table 7). In this sense, “center-periphery = 0.585,” which means that, at the time of the assessment, students in the center of Granada obtained 0.585 more correct answers (on average) than those in the periphery. In terms of the school year, “4th CSE - 1st CSE = 1.483,” which means that, in the average score obtained at that time, 4th CSE obtained 1.483 more correct answers (on average) than 1st CSE. Finally, in relation to gender, “women - men = 1,483” means that, at the time of the assessment, women obtained 1,483 more correct answers (on average) than men.

**Table 7:** Comparison of results in the evaluation carried out after the training intervention (T1) according to the 3 variables analyzed.

Comparison (first–second)	Difference	CI 95% (lower; upper)	P
Center–Periphery	0.585	0.027–1.196	0.061
4th CSE−1 st CSE	1.483	0.884–2.082	0.000
Female–Male	1.483	0.818–1.988	0.000

The values indicated correspond to contrasts of estimated marginal means (EMMs).

Finally, Table 8 shows that the degree of knowledge retention was higher in schools located in the city of Granada, among students in the upper grade, and among women. Thus, “center-periphery = 2,523,” which means that, at the time of the assessment, students at the center in Granada obtained 2,523 more correct answers (on average) than those in the periphery. As for the school year, “4th CSE - 1st CSE = 2,284,” which means that, in the average score obtained at that time, 4th CSE obtained 2,284 more correct answers (on average) than 1st CSE. Finally, in relation to gender, “women - men = 0.702” means that, at the time of the assessment, women obtained 0.702 more correct answers (on average) than men.

**Table 8:** Comparison of results in the evaluation 6 months after the training intervention (T2) according to the 3 variables analyzed.

Comparison (first–second)	Difference	CI 95% (lower; upper)	P
Center–Periphery	2.523	1.978–3.067	0.000
4th CSE−1 st CSE	2.284	1.729–2.840	0.000
Female–Male	0.702	0.150–1.253	0.013

The values indicated correspond to contrasts of estimated marginal means (EMMs).

## 4 Discussion

A study was conducted initially involving 459 students from six educational centers (three located in the city of Granada and three in its metropolitan area/suburbs), of whom 420 completed the study. According to the results obtained in our study, the students generally showed a notable lack of knowledge of CPR maneuvers before the intervention (initial average percentage of correct answers around 40%), which after the training intervention increased to just over 67%. 6 months after the training intervention, this knowledge suffered the effect of the forgetting curve, with the average percentage falling to 57%. The age of the intervention group in this study ranged from 12 years old (1st-year CSE students, who accounted for 45.7% of the total) to 15 years old (4th-year CSE students, accounting for the remaining 54.3%), with the older students obtaining the highest average score in the three assessments. There was equal participation in terms of student gender (49.5% female and 50.5% male), with a significant difference in favor of females in the average results obtained in the assessment carried out immediately after the intervention (T1) and after 6 months (T2). A distinction was also made according to the location of the educational center where the students were studying (52.4% in centers in the capital and 47.6% in centers in the suburbs), with students in the capital obtaining higher average results in T1 and T2.

After the intervention, in which the importance of CPR and early initiation of CPR was explained and the students were able to practice these maneuvers, the percentage of correct answers improved to 67.38%, which is consistent with the results obtained in studies conducted in schoolchildren (3, 5, 6, 11, 36)^.^

When analysing differences by age/grade, we observed an improvement after the intervention for all students, but with higher gains and retention for students in more advanced grades. This relationship is consistent with the school literature, where age is associated with a greater ability to integrate decision sequences, sustain attention during structured tasks and consolidate complex learning; in addition, from 10–12 years of age, the probability of performing compressions with parameters closer to the recommended ones increases, which enhances the significant learning of procedural components (37). Consequently, the differences between age ranges described in previous studies can be interpreted not only as an ‘older age' effect, but as reflecting two domains with distinct trajectories: (i) cognitive and attitudinal content that can be introduced early (safety, emergency system activation, recognition of abnormal breathing) and (ii) motor skills that are optimised as physical development progresses (37). This evidence supports a progressive curricular approach: start as early as possible with the first links and reinforce them periodically, gradually incorporating more physically demanding skills and scenarios, which is in line with the international ‘Kids Save Lives' framework, which recommends starting no later than age 12 and maintaining recurrent training (11, 38–41).

**Figure 2:**
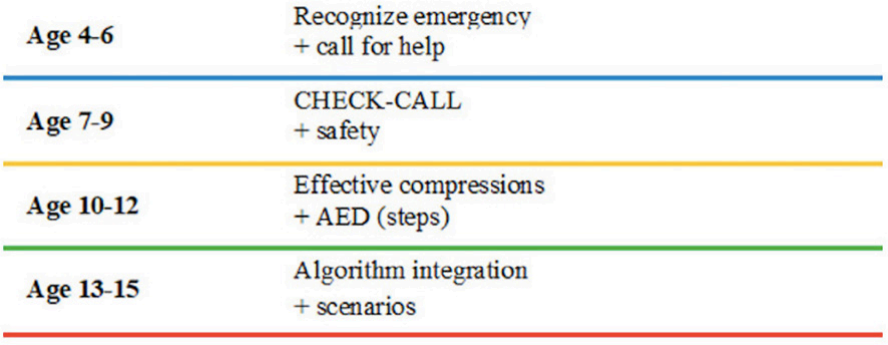
Progression by age (school-based SVB) and relationship with retention.

On the other hand, there is controversy regarding the age at which BLS training should begin. The ERC sets the starting age at 12, while the AHA recommends that it be mandatory from the age of 9 (6). However, several studies reinforce the idea of starting such training at an earlier age (even in early childhood education) (1, 42), introducing concepts and skills gradually. According to self-efficacy theory, it has been proven that younger schoolchildren are capable of internalizing knowledge and carrying out actions related to life support (e.g., memorizing the emergency telephone number and making a call to alert someone), and this has also been shown to reduce anxiety about making mistakes, increase empathy and willingness to help others, and when they reach the age, build, and maturity to perform quality chest compressions, they have retained the necessary knowledge and skills, obtaining better results than other schoolchildren who began their training later (5, 6, 11). All of this promotes a “culture of CPR” as a basic conceptual element for any society. Figure 2 visually shows the progression by age of school SVB content and its relationship to retention.

**Figure 3:**
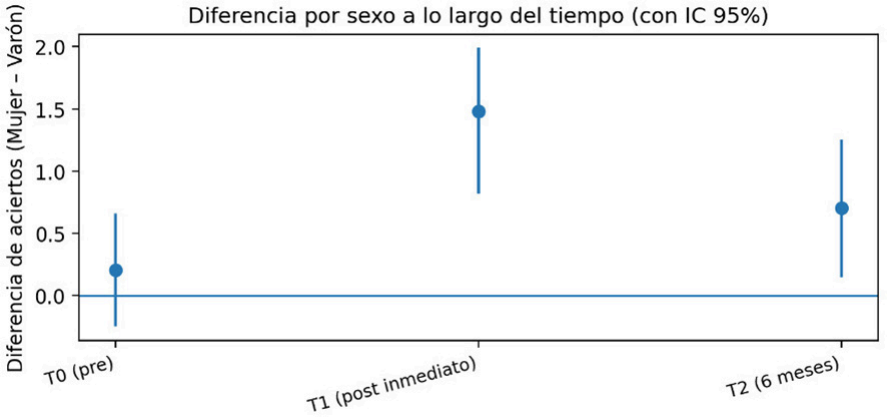
Difference by gender over time (with 95% CI).

In our study, females showed higher scores than males mainly after the intervention (T1), with the difference attenuating at follow-up (T2). (3, 5, 17). This pattern suggests that the effect may be related to differences in response to learning and/or consolidation, rather than to greater baseline knowledge. We interpret this finding with caution, given that gender may act as a marker for contextual variables (course, school, participation in internships, self-efficacy or assessment anxiety). From an applicable point of view, these results reinforce the desirability of instructional designs that ensure active participation and role rotation, deliberate practice with observable criteria and brief reinforcement (microlearning), so that the programme maximises acquisition and retention for all learner. Figure 3 shows a simple graph of the female-male difference and its 95% CI at T0-T1-T2.

The University of Granada has carried out various training activities in BLS in schools in the capital, which is why we analyzed whether there were differences between students according to the location of the school where they studied (Granada capital vs. the metropolitan area). In this regard, significant differences were only observed in the results of the third assessment in favor of students from schools in the capital; however, it is difficult to compare this result with other studies, as we have not found any literature that allows us to do so, and further research is needed to find an explanation that justifies it.

**Figure 4:**
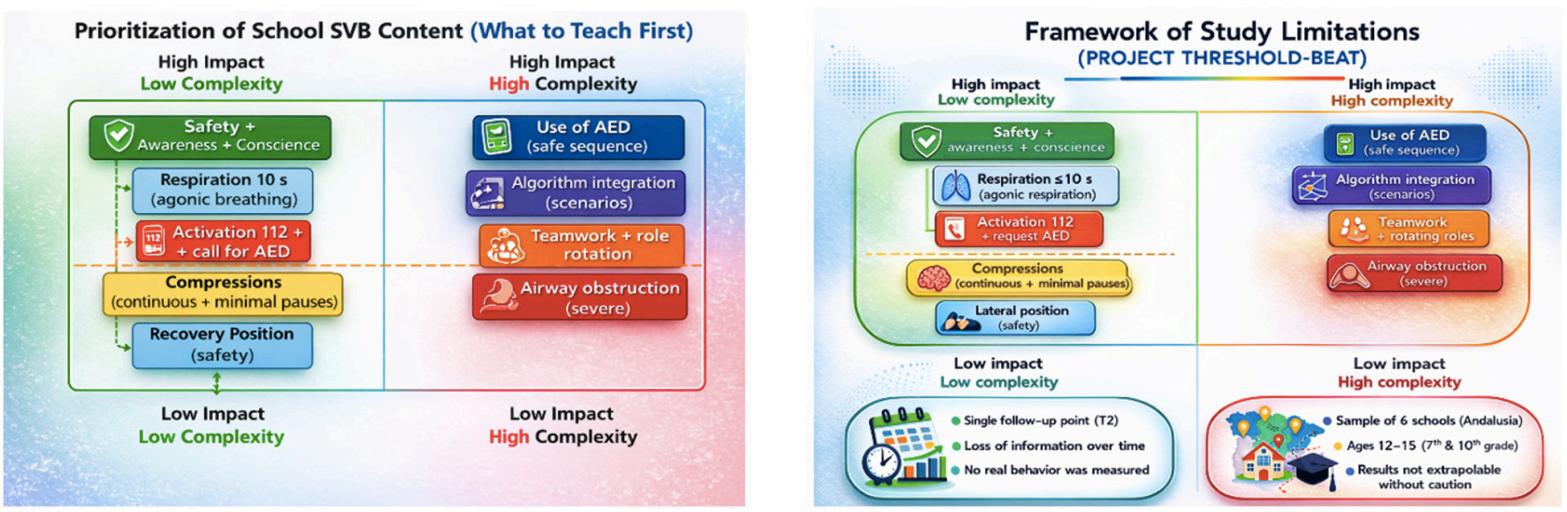
Prioritisation figure based on impact and complexity.

Beyond who acts as trainer, a determining aspect for the population effectiveness of school CPR is what content is prioritised and in what sequence it is introduced. In line with our results and with previous evidence, we propose a minimum core of school BLS focused on high-impact and high-retention contents: safety and recognition of unconsciousness, rapid assessment of breathing (including agonal breathing) in ≤ 10 s, early activation of the emergency system and request for AED, and immediate initiation of continuous chest compressions minimising interruptions. On top of this core, and depending on age and time available, high-priority content such as safe AED use and algorithm integration in brief scenarios are added, as well as complementary content such as severe airway obstruction and the safe lateral position. This approach allows maximising initial acquisition and targeting periodic reinforcement (microlearning) to the components most fragile to loss, favouring medium-term retention. Table 9 and Figure 4 show the prioritized curriculum based on impact and complexity.

**Table 9:** Proposed priority content.

Priority	Content	Observable objective	Recommended assessment
1 (essential)	Safety and recognition of unconsciousness	Identifies safe scene and unresponsiveness	Item + mini-checklist
1 (essential)	Assessment of breathing ≤ 10 s (incl. agonal breathing)	Decide ‘not breathing normally' without delay	Key item
1 (essential)	Activation of emergency system (112) and request AED	Call/indicate call and request AED	Item + scenario
1 (essential)	Continuous quality chest compressions (minimal interruptions)	Initiate compressions and maintain continuity	Checklist CPR
2 (high priority)	Use of AED (safe sequence)	Patches and follows prompts without long pauses	AED Checklist
2 (high priority)	Integration of the complete algorithm (short scenario)	CHECK-CALL-COMPRESS-DEA Sequence	Scenario + rubric
3 (complementary)	Severe airway obstruction	Identifies severity and applies sequence	Item + role-play
3 (complementary)	Lateral safety position	Correctly position if breathing	Short checklist

The knowledge, skills, and abilities required for life support, as in other subjects, are subject to the forgetting curve described by Ebbinghaus, such that if this knowledge is not used regularly, it will gradually be forgotten. In this regard, our study observed a decline in knowledge 6 months after the intervention, with half of the key concepts being forgotten, which is consistent with the results obtained in similar studies (13). This forgetting curve is the reason why the ERC and AHA recommend annual training (5–7, 12, 25–32, 43). Given the gradient of forgetting observed at 6 months and considering the evidence on spaced practice and microlearning, we propose that reinforcement interventions should not be spaced beyond 6 months. In this sense, the implementation of short, targeted micro-refresher updates on a quarterly basis could contribute to consolidate retention, although future studies should comparatively evaluate different reinforcement intervals.

Various studies have analyzed training with theoretical-practical interventions vs. purely theoretical interventions, with better results obtained in the former (1). We therefore recommend training with theoretical-practical interventions whenever possible, as it is essential to acquire procedural and attitudinal skills in order to correctly carry out all the actions included in BLS.

In this quasi-experimental study, implementation of the structured formative model was associated with significant improvement in knowledge and competence scores immediately after the intervention, with partial maintenance at 6 months. These results suggest that a curriculum approach focused on priority content and reinforced by periodic micro-interventions can contribute to consolidating learning in secondary school students.

However, given the non-randomised design and the limited follow-up period, our findings should be interpreted with caution. The study does not allow us to establish definitive causal relationships or to directly extrapolate the results to other educational contexts without further validation. Future controlled studies with longer follow-up will be necessary to confirm the stability of the observed effects and to assess their impact on behavioural or community indicators.

Taken together, our data support the feasibility and potential pedagogical utility of the proposed model in school settings similar to the one evaluated, rather than a claim of universal or definitive efficacy.

**Figure 5:**
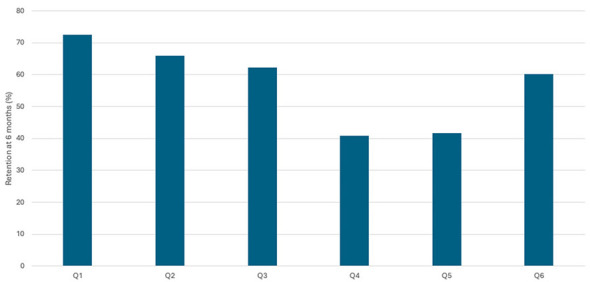
Retention by item (Q1–Q6).

A content-level finding is that item Q1 showed higher retention at 6 months than the other key questions. This difference suggests that the components of the school SVB do not exhibit a uniform forgetting trajectory. We interpret the greater stability of Q1 as compatible with its lower cognitive load and regimented nature, its greater prior exposure to public health messages, and its high perceived relevance. In contrast, items requiring fine discrimination or complex sequences tend to be more vulnerable to forgetting and would benefit from periodic targeted micro-reinforcement. Table 10 provides analytical and psychometric support, and Figure 5 shows the differential retention at 6 months, which guides curriculum prioritization based on “fragile content”.

**Table 10:** Analytical and psychometric support.

Aspect	What to check in Q1	How we will report it
Item difficulty	Higher proportion of hits on T1/T2	p(T0/T1/T2) + absolute changes
Discrimination	Adequate discrimination or stability	Item-total correlation/discrimination index
Longitudinal model	Item × time interaction	Mixed model with item factor + MMEs
Forgetting	Lower drop T1 → T2	Δ(T1–T2) per item with 95%CI

### 4.1 Limitations and generalisation considerations

**Figure 6:**
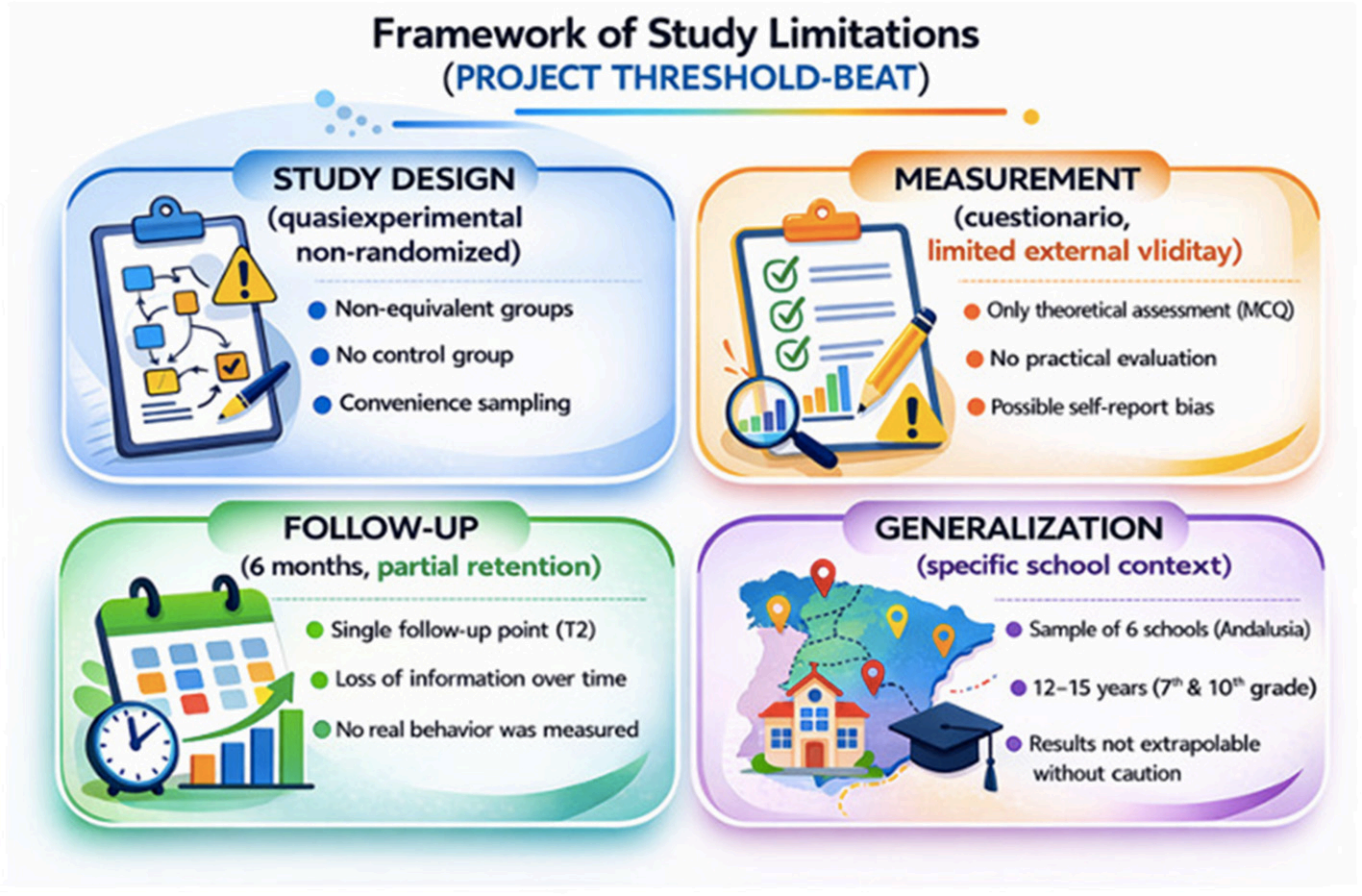
Framework of study limitations.

This study has several limitations that should be considered when interpreting the results (Figure 6). First, the quasi-experimental design without random assignment limits the ability to establish causal relationships. Although multivariate analysis and mixed models were applied to adjust for potential confounders, the presence of residual confounding cannot be ruled out.

Second, outcome measurement was based on a structured questionnaire and practical assessments in a simulated environment. While these instruments allow estimation of knowledge and skills acquisition and retention, they do not assess actual performance in a cardiopulmonary arrest in a community setting.

Third, follow-up was limited to 6 months. Although partial maintenance of knowledge was observed, long-term stability cannot be inferred without further assessment beyond this period.

Fourthly, the sample comes from a specific educational context, with specific socio-cultural and organisational characteristics. Therefore, generalisation of the results should be made with caution to other education systems or international settings with different curricular structures or available resources.

Finally, although the study analyses differences by grade and gender, other psychosocial determinants (e.g., motivation, self-efficacy or school climate), which might modulate the response to the intervention, were not explored in depth.

Overall, our findings should be interpreted as contextual evidence on the feasibility and association of the formative model with improvements in knowledge and competence in the assessed setting, rather than as definitive proof of universal efficacy.

## 5 Conclusions

In this quasi-experimental study conducted with secondary school students, the structured formative intervention was associated with significant improvement in performance immediately after training and partial maintenance at six months. These findings support the feasibility of the model and suggest that retention may decline over time, justifying consideration of periodic reinforcement strategies.

However, the optimal refreshment interval (e.g., quarterly vs. half-yearly), the minimum starting age (primary vs. secondary) and the effect of different teacher training strategies were not directly assessed in this study. Therefore, these proposals should be interpreted as reasonable hypotheses for future research, rather than as conclusive recommendations. Future comparative studies, ideally controlled and with longer follow-up, should specifically examine the periodicity of refreshers, early start of the curriculum and train-the-trainer models, as well as their impact on behavioural and performance indicators in real contexts.

## Data Availability

The datasets presented in this study can be found in online repositories. The names of the repository/repositories and accession number(s) can be found below: https://drive.google.com/drive/folders/16CmhkmEkI8cHgOc3sVbYCAbbceh6v3jd.
