# Supplementary material for: Crossing life-saving thresholds: learning-forgetting trajectories in secondary-school basic life-support training: project threshold-beat
Source: Front Public Health. 2026 May 7;14:1777690. doi: 10.3389/fpubh.2026.1777690 (PMC13190590; doi:10.3389/fpubh.2026.1777690)
Supplement: Supplementary file 1 [file Supplementary_file_1.pdf]

Supplementary Material

1 Supplementary Data

Completed GREET checklist

**Completed GREET checklist**			
**GREET item**	**Description required**	**Location in revised manuscript**	**Comments**
**Context**	Educational environment and setting	Methods - Context (p.8-9, lines 189-201)	Schools, educational level, school setting
**Participants**	Inclusion/exclusion criteria	Methods - Participants (p.9, lines 202-208)	Total number, losses and follow-up
**Trainers**	Profile and training	Methods - Trainers (p.9, lines 209-221)	Calibration and standardisation session
**Content**	Themes and training objectives	Methods - Intervention (p.9, lines 222-227)	Detailed table of components
**Materials**	Resources used	Methods - Intervention (p.9-10, lines 228-244)	Dummies, AEDs, presentations
**Teaching methods**	Teaching strategies	Methods - Intervention (p.10, lines 245-256)	Deliberate practice + microlearning
**Dosage**	Duration and intensity	Methods - Schedule (p.10, lines 257-263)	T0, T1, T2 and micro-refreshers
**Evaluation**	Instruments and reliability	Methods - Instruments (p.10-11, lines 264-274)	KR-20 and evaluation schedule
**Analysis**	Statistical model	Methods - Analysis (p.11, lines 275-285)	Specified mixed model

Note: Final pages and lines will be adjusted after final layout according to proofs.

In review

Participant flow diagram

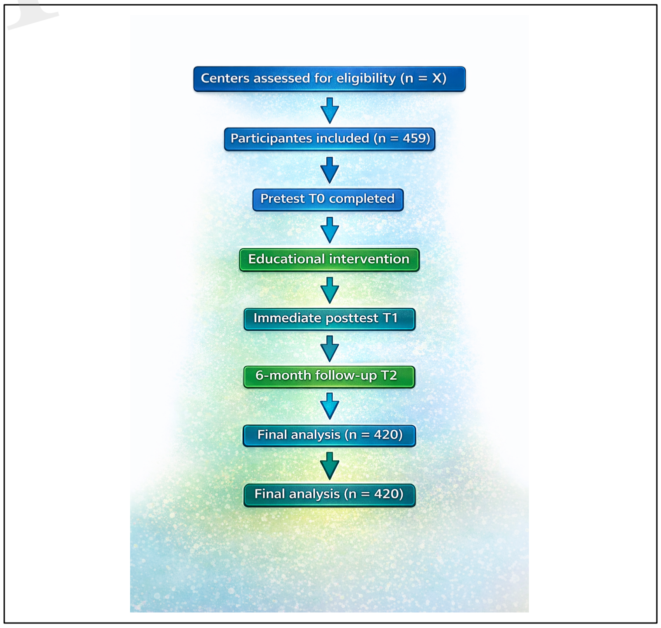

Curriculum table

Curriculum table						
**Module**	**Objective**	**Method**	**Time**	**Material**	**Key elements addressed**	**Assessable output**
**M1. Introduction/safety**	Aligning safe behaviour and quick decision	Pre-briefing + mini-class	10-15 min	Slides + script	1,4	Pretest/items
**M2. CHECK-CALL**	Recognise PCR and activate 112/DEA	Demonstration + guided practice	15-20 min	Cards/script	1,3,4	Partial checklist
**M3. COMPRESS**	Quality compressions and continuity	Deliberate practice	25-35 min	Dummy	2,5	CPR checklist
**M4. AED**	Safe use of the AED and minimisation of pauses	Station + role-play	15-20 min	AED training	3,5	AED checklist
**M5. Integrated scenario**	Complete sequence under pressure	Short simulation	10-15 min	Scenario script	1-5	Overall performance
**M6. Choking**	Identify severe obstruction and take action	Demonstration + role-play	10-15 min	Script	6	Key item
**M7. Debriefing**	Consolidation and self-efficacy	Structured debriefing	10 min	Guide	1-6	Cognitive reinforcement
**Micro-refresher**	Active spaced retrieval	Microlearning	5-10 min	QR/LMS	priority	Mini-test

In review

Curriculum map

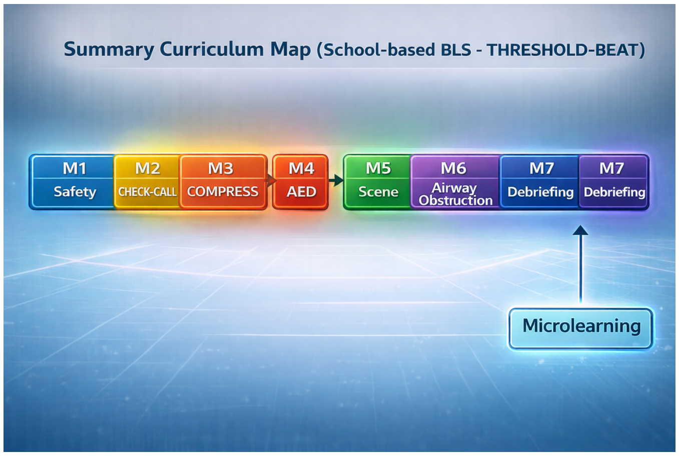

Graphical presentation of results using forest plots

Figure - T0

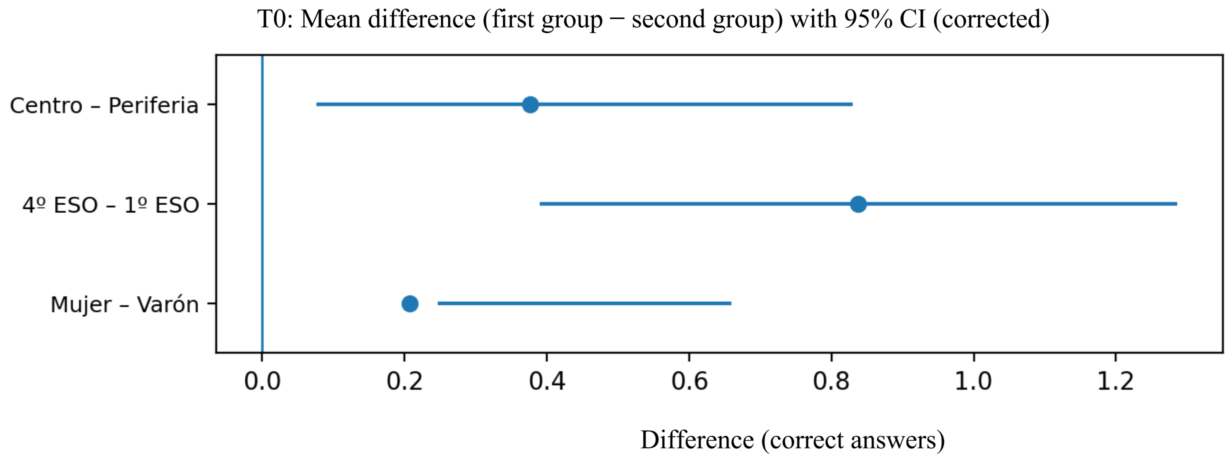

Figure - T1

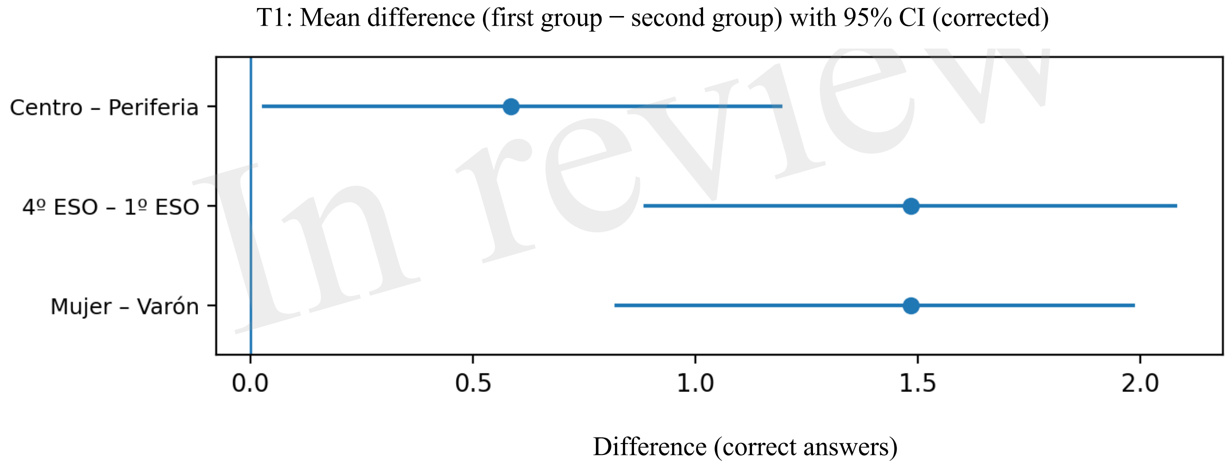

In review

Figure - T2

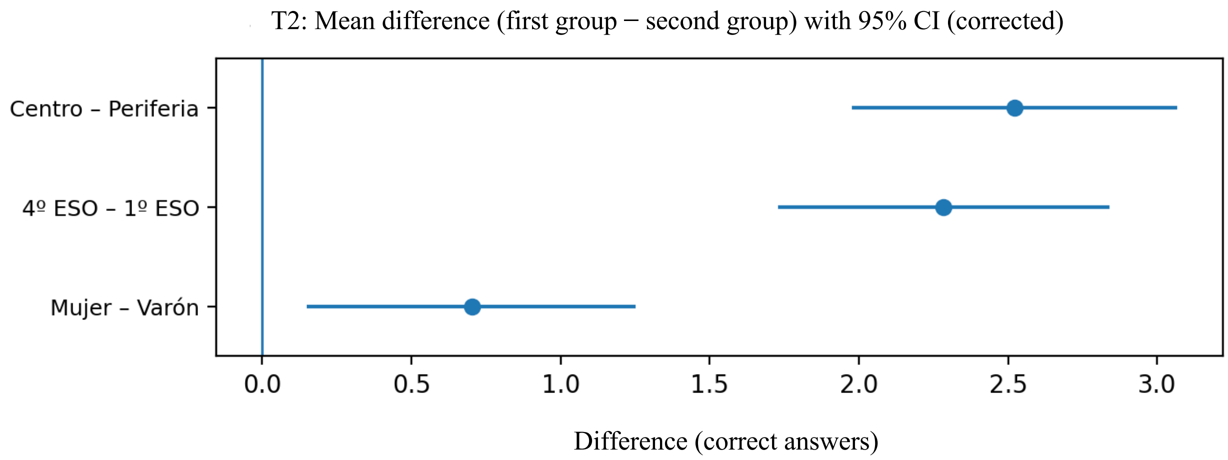

Figure 7. Summary image of the project *"Crossing life-saving thresholds: learning–forgetting trajectories in secondary-school basic life-support training"*. This figure provides a visual overview of the main and most relevant elements of the article, highlighting the key components of the study design, intervention, and learning–forgetting processes in basic life

support training among secondary school students.

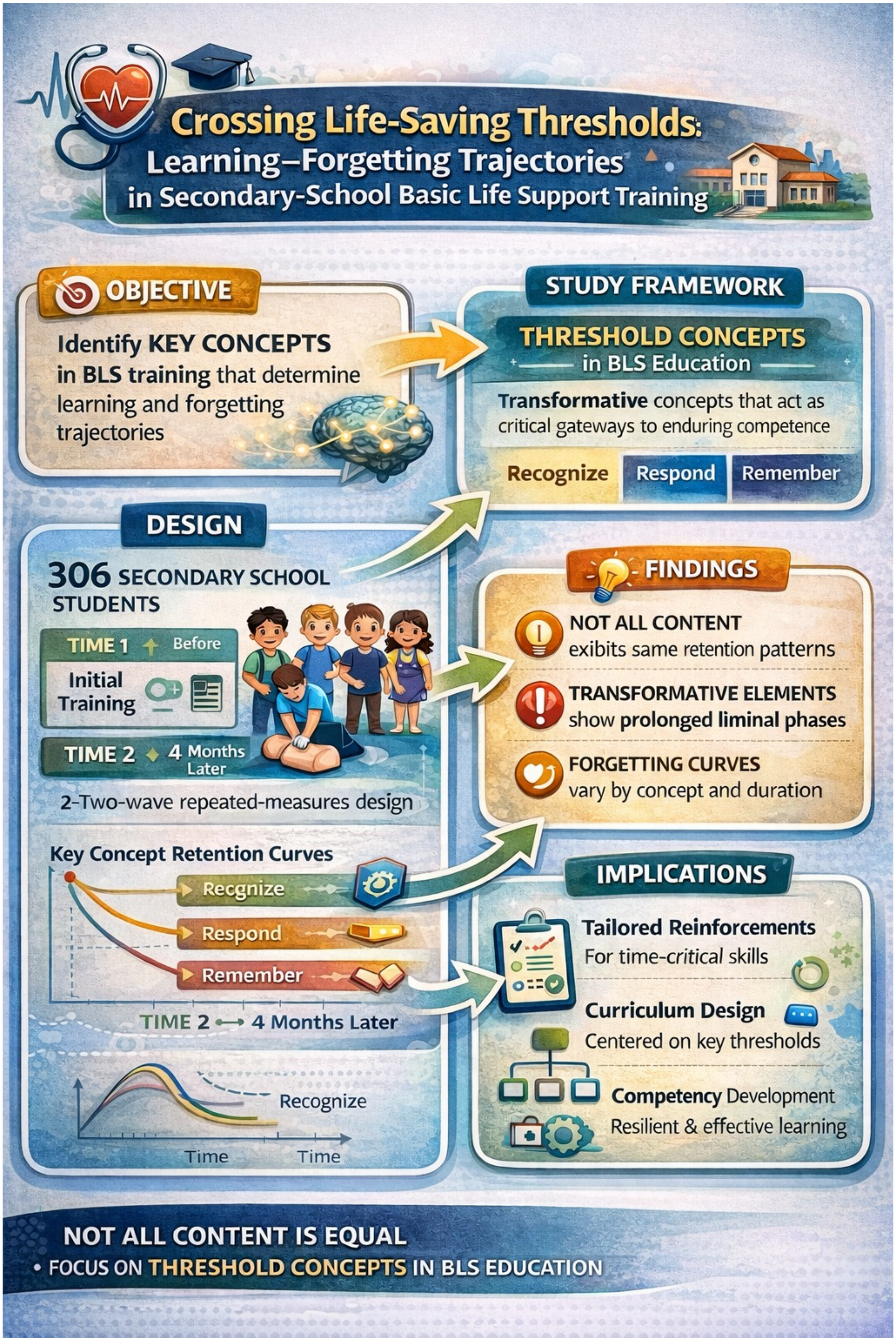

Crossing Life-Saving Thresholds:
Learning–Forgetting Trajectories
in Secondary-School Basic Life Support Training
OBJECTIVE
Identify KEY CONCEPTS in BLS training that determine learning and forgetting trajectories
STUDY FRAMEWORK
THRESHOLD CONCEPTS
in BLS Education
Transformative concepts that act as critical gateways to enduring competence
Recognize
Respond
Remember
DESIGN
306 SECONDARY SCHOOL STUDENTS
TIME 1 Before
Initial Training
TIME 2 4 Months Later
2-Two-wave repeated-measures design
Key Concept Retention Curves
Recgnize
Respond
Remember
TIME 2 4 Months Later
Recognize
Time
Time
FINDINGS
NOT ALL CONTENT
exibits same retention patterns
TRANSFORMATIVE ELEMENTS
show prolonged liminal phases
FORGETTING CURVES
vary by concept and duration
IMPLICATIONS
Tailored Reinforcements
For time-critical skills
Curriculum Design
Centered on key thresholds
Competency Development
Resilient & effective learning
NOT ALL CONTENT IS EQUAL
• FOCUS ON THRESHOLD CONCEPTS IN BLS EDUCATION
